# Lipid profiles in French West Indies sickle cell disease cohorts, and their general population

**DOI:** 10.1186/s12944-018-0689-5

**Published:** 2018-03-05

**Authors:** Marie-Laure Lalanne-Mistrih, Philippe Connes, Yann Lamarre, Nathalie Lemonne, Marie-Dominique Hardy-Dessources, Vanessa Tarer, Maryse Etienne-Julan, Dominique Mougenel, Benoît Tressières, Marc Romana

**Affiliations:** 1 Université des Antilles, CHU de Pointe-à-Pitre, Guadeloupe, Université Sorbonne Paris Cité, Université Paris Diderot, Inserm, INTS, Unité Biologie Intégrée du Globule Rouge UMR_S1134, laboratoire d’Excellence GR-Ex, Paris, France; 2 Centre d’investigation Clinique Antilles Guyane, Inserm/DGOS CIC 14-24, enceinte de l’Institut Pasteur, Pointe-À-Pitre, Guadeloupe France; 3 Institut Universitaire de France, Paris, France; 4 Laboratoire LIBM EA7424, Equipe « Biologie Vasculaire et du Globule Rouge », Laboratoire d’Excellence GR-Ex, Université de Lyon, Lyon, France; 5 Unité Transversale de la Drépanocytose, CHU de Pointe à Pitre, Pointe-à-Pitre, Guadeloupe France; 6 UMR Inserm 1134, Hôpital Ricou, CHU de Pointe-à-Pitre, 97 157 Pointe-à-Pitre, Guadeloupe France

**Keywords:** Genetics, Epidemiology, Triglycerides, Lipoproteins, Apolipoproteins, Sickle cell disease, Body mass index, Abdominal obesity, Non HDL-cholesterol, Osteonecrosis

## Abstract

**Background:**

The pathophysiology of sickle cell disease (SCD) and the variability of its clinical expression remain not fully understood, whether within or between different SCD genotypes. Recent studies have reported associations between lipid levels and several SCD complications. If lipid levels have been previously described as low in sickle cell anemia (SCA), few data have been provided for sickle cell SC disease (SCC). We designed our epidemiological study to isolate lipid levels and profiles by genotype in Guadeloupian cohorts of SCA and SCC adult patients, at steady state. We compared SCD lipid levels with those of the Guadeloupian general population (GGP), and analyzed potential associations between lipid levels and SCD complications (vaso-occlusive crises, acute chest syndrome and osteonecrosis).

**Methods:**

Lipids, apolipoproteins, biological variables and anthropometric evaluation, were collected at steady state from medical files for 62 SCC and 97 SCA adult patients. Clinical SCD complications were collected from the clinical files. Analysis was conducted by genotype for all variables.

**Results:**

Different SCC and SCA lipid profiles, both distinct from their GGP’s, were identified. Compared to SCC and GGP, higher triglyceride (TG) levels were observed in SCA patients, independent of hydroxyurea, hemolysis, gender, age, body mass index (BMI), abdominal obesity and clinical nutritional status. Our survey highlights also subsequent anthropometrical phenotypes, with an over-representation of abdominal obesity with normal BMI in SCA patients, and affecting almost exclusively females in both genotypes. Moreover, more frequent positive history of acute chest syndrome (ACS) was observed in SCA patients with TG level higher than 1.50 g/l, and of osteonecrosis in SCC patients having non high-density lipoprotein-cholesterol level (Non HDL-C) higher than 1.30 g/l.

**Conclusions:**

This study reveals that SCA and SCC patients exhibit distinct lipid profiles and suggests that high TG and Non HDL-C levels are associated with past histories of ACS and osteonecrosis in SCA and SCC patients, respectively.

## Background

Sickle cell disease (SCD) is a monogenetic disorder resulting from a point mutation in the β-globin gene leading to the synthesis of abnormal hemoglobin S (HbS). Sickle cell anemia (SCA), i.e. the homozygous state of the β^S^ allele, is the most frequently encountered genotype worldwide, far beyond sickle cell SC disease, i.e. the heterozygous composite state of the β^S^ and β^C^ alleles (SCC). The sickling of red blood cells (RBCs), due to polymerization of HbS when deoxygenated, is the main pathophysiological mechanism at the origin of several vaso-occlusive-like events resulting from the entrapment of poorly deformable and fragile sickle red blood cells in small vessels [1]. SCD is characterized by chronic hemolysis, inflammation, exacerbated oxidative stress, frequent vaso-occlusive complications, multiple organ damage and reduced patient survival [1]. There are large variations in the nature and incidence of complications affecting SCA and SCC patients, and the clinical severity of SCC is often considered to be milder than that of SCA [2]. By contrast, it has been recently shown that SCC patients may also frequently experience similar vaso-occlusive-like events than SCA patients, i.e. vaso-occlusive crisis (VOC), acute chest syndrome (ACS) and osteonecrosis (OTN), and may also develop more frequently specific complications such as retinopathy and otologic disorders [3, 4]. The exact pathophysiological mechanisms, which lie at the origin of the heterogeneous clinical severity in SCC and SCA patients, have yet to be fully elucidated.

Lipids have been very recently hypothesized to play a role in the pathophysiological mechanisms of SCA. SCA patients have unique plasma lipid profile characterized both in adults and children, by decreased levels of total cholesterol (TC), high density lipoprotein-cholesterol (HDL-C), low density lipoprotein-cholesterol (LDL-C), apolipoprotein A (apoA), and apolipoprotein B (apoB), compared to controls or to the general population [5–8]. Contrasting results have been reported regarding the level of triglycerides (TG) in SCA compared to controls [7, 9, 10]. This dyslipidemia has been associated with the severity of hemolysis and would be involved in vascular dysfunction [7, 11, 12]. In addition, it has been also shown that patients with the highest TG levels would be prone to develop complications like pulmonary hypertension [7, 11, 12] and acute chest syndrome [13]. Furthermore, two recent studies showed evidence of an enhanced production of deleterious pro-inflammatory HDL-C that has been associated with endothelial cell injury in SCA patients [14, 15]. Altogether, these data support a deleterious impact of dyslipidemia on endothelial cells function in SCA patients and suggest that alterations in lipids profile could be modulate or reflect the disease severity. Yet, no precise information about lipid profile and their role in the pathophysiology is currently available for SCC adult patients. Unless recently [14, 16], the rare studies including both SCA and SCC patients did not analyze lipids according to sickle genotypes [7, 9, 10].

The major aim of the present study was to characterize blood lipid profiles in SCA and SCC adult patients, at steady state, and to compare them to those of the Guadeloupian general population (GGP). In addition, we also analyzed, still according to genotypes, the associations between blood lipids, routine clinical parameters and the history of several vaso-occlusive like complications (VOC, ACS and OTN).

## Methods

### Subjects

One hundred and fifty nine sickle cell adults (62 SCC and 97 SCA) at steady state, regularly monitored and followed up by the sickle cell reference center of Guadeloupe, were included in the present study between May 2010 and December 2011. The SCC/SCA sex ratio (M/F) was 0.67, with a mean age of 36 ± 13 years old.

The inclusion criteria were previously detailed [12]. The steady-state condition was defined as follows: no blood transfusion in the previous three months, and absence of acute episodes (infection, VOC, ACS, stroke, priapism) at least two months before inclusion into the study. Twenty-one of 95 SCA patients (i.e., 22.1%) were undergoing hydroxyurea (HU) therapy. None of SCC patients was under HU treatment. All patients had been informed about the purpose and procedures of this study, for which they had given a written consent in accordance with the guidelines set by the Declaration of Helsinki. The study was approved by the Regional Ethics Committee (CPP Sud/Ouest Outre Mer III, Bordeaux, France, registration number: 2010-A00244-35).

### Anthropometric parameters

Patients underwent a complete anthropometric examination. Height (cm) and weight (kg) were measured for all patients and BMI was calculated as the ratio of weight (kg)/height^2^ (m^2^). Selected BMI classes were BMI < 18.5 kg/m^2^ (underweight), 18-24.9 kg/m^2^ (normal corpulence), ≥25-29.9 kg/m^2^ (overweight), ≥30 kg/m^2^ (obesity). The definition of undernutrition or malnutrition was based on the following clinical items: BMI < 18.5 kg/m^2^ in adults of less than 70 years old or of BMI < 21 kg/m^2^ in adults of more than 70 years old. Waist circumference was measured according to the NHANES III protocol [17]. Waist-to-hip ratio (WHR) was calculated from waist and hip circumferences measurement [18]. We chose, as abdominal obesity definition, those following the International Diabetes Federation criteria (waist> 94 cm male; waist> 80 cm female) [19].

### Controls

Control values for TC, HDL-C, LDL-C, TG and BMI of the GGP were issued from a previous study [20]. GGP control values of abdominal obesity and BMI classes available for the same period as in our study were issued from Daigre et al. [21].

### Biological measurements

Blood samples were collected after 12 h of overnight fasting and analyzed at the University Academic Hospital of Pointe-à-Pitre (Guadeloupe). Hemoglobin concentration (Hb), hematocrit, reticulocytes (RET), red blood cell counts (RBC), platelet counts (PLT), and white blood cell counts (WBC) were determined using a hematology analyzer (Max M-Retic, Coulter, USA). The dosages for fasting glycemia, total bilirubin (BIL), lactate dehydrogenase (LDH) and aspartate amino-transferase (AST) were performed using standard biochemistry. The measurements of TC, HDL-C, LDL-C and TG were performed using a colorimetric method (Integra Cobas Roche) and those of apoA and apoB with a nephelometric method (Behring Nephelemeter 2, Siemens). LDL-C was calculated according to the Friedwald formula: LDL-C (g/l) = TC (g/l) – HDL-C (g/l) – TG/5 (g/l). Non-HDL cholesterol (Non HDL-C) was calculated using the following formula: Non HDL-C (g/l) = TC (g/l) – HDL-C (g/l).

### SCD complications

The previous occurrence of OTN, ACS and hospitalized painful VOC were collected from patients’ medical files at the sickle cell reference center of Guadeloupe. The collection of OTN history started with the very first retrospective patient’s follow-up at the SCD reference center while for VOC and ACS, it started one year before the annual patients’ checkup. OTN, ACS and hospitalized VOC diagnoses were established as previously described [22–25].

### Statistical analysis

Results are presented as means ± Standard deviation (SD) for continuous variables and as frequencies for categorical variables. To compare co-variables between different groups, unpaired Student’s t-test was used for continuous covariates, and chi-square-test or exact Fisher Test, for categorical covariates. Pearson test was used to investigate correlations. A principal component analysis was used to derive a hemolytic component value from the four hemolytic markers being investigated (i.e. BIL, LDH, AST and RET), as previously reported [26]. This standard statistical data reduction approach employs conventional clinical measurements to explain the maximum-shared variance among these indirect measures of hemolysis. Following 2011 ESC/EAS European guidelines [27], thresholds of 1.30 g/L for Non HDL-C and of 1.50 g/L for TGL were chosen, to explore the links with the SCD complications observed.

The significance level was defined as *p <* 0*.*05. Analyses were conducted using SPSS (v. 20, IBM SPSS Statistics, Chicago, IL).

## Results

### SCC and SCA corpulence distributions vs. GGP

Results are reported in Tables 1 and 2. SCC and SCA patients presented similar values for the following parameters: sex ratio, age, waist, WHR and abdominal obesity. Mean BMI was not different between SCC patients and GGP, and was lower in SCA vs. SCC patients and GGP (*p* < 0.01). The undernutrition class was significantly over-represented in SCA patients compared to GGP (*p* < 0.01) (Table 2), with no difference detected between SCC patients and GGP. If overweight was less frequently encountered in SCA patients compared to GGP and SCC patients (*p* < 0.01 for both), obesity remained low and similar between SCA and SCC patients, and much lower than in GGP (*p* < 0.01 for both, Table 2). Abdominal obesity affected exclusively females (with the exception of one SCC male), with a lower prevalence for both genotypes than in GGP (*p* < 0.01 for both, Table 2), and was undetected in the undernutrition class in both genotypes (data not shown).

**Table 1:** Lipid levels and biological characteristics in SCC and SCA cohorts versus Guadeloupian general population^a^

	GGP^a^	SCC	SCA
	*N* = 1010	N_1_ = 62	N_2_ = 97
Sex ratio (M/F)	0.66	0.51	0.80
Age (years)	39.5 ± 13.3	37.5 ± 13.3	34.6 ± 12.9^**^
BMI (Kg/m^2^)	25.7 ± 5.3	24.5 ± 4.5	21.5 ± 3.3^** °°^
Waist (cm)	88.9 ± 16.6	81.6 ± 10.2^**^	79.0 ± 7.1^**^
WHR (cm/cm)	0.81 ± 0.91	0.85 ± 0.07	0.86 ± 0.05^**^
Systolic arterial tension (mmHg)	127.8 ± 18.1	120.2 ± 14.6^**^	114.4 ± 10.6^** °^
Diastolic blood pressure (mmHg)	81.9 ± 12.8	74.5 ± 7.4^**^	67.1 ± 7.4^** °°^
Hb (g/dl)	–	11.3 ± 1.2	8.4 ± 1.3^°°^
Hemolytic Component	–	- 0.880 ± 0.189	0.556 ± 0.712^°°^
TC (g/L)	1.90 ± 0.43	1.40 ± 0.32^**^	1.21 ± 0.29^** °°^
HDL-C (g/L)	0.48 ± 0.13	0.44 ± 0.12^*^	0.37 ± 0.10^** °°^
Apo A (g/l)	–	1.24 ± 0.20	1.11 ± 0.20^°°^
LDL-C (g/L)	1.26 ± 0.39	0.79 ± 0.24^**^	0.66 ± 0.23^** °°^
Apo B (g/l)	–	0.66 ± 0.19	0.60 ± 0.18
Non HDL-C (g/l)	1.41 ± 0.41	0.96 ± 0.30^**^	0.85 ± 0.26^**°^
TG (g/L)	0.79 ± 0.45	0.80 ± 0.43	0.97 ± 0.39^** °^
TC / HDL-C	4.1 ± 1.3	3.31 ± 0.98^**^	3.46 ± 0.86^**^
TG / HDL-C	–	2.11 ± 1.96	2.93 ± 1.79^°°^
Fasting glycemia (g/l)	0.90 ± 0.31	0.75 ± 0.17^**^	0.72 ± 0.11^**^

Values represent mean ± standard deviation; comparison with GGP (Guadeloupean general population): ^*^p < 0.05; ^**^p < 0.01; comparison with SCC population: ^°^*p* < 0.05; ^°°^*p* < 0.01; ^a^Data issued or calculated (Non HDL-C) from reference (18)

**Table 2:** BMI classes and abdominal obesity in SCC and SCA cohorts versus Guadeloupian general population ^a^

	GGP^a^	SCC	SCA
	*N* = 602	N_1_ = 62	N_2_ = 97
BMI < 18.50 (kg/m^2^) Undernutrition class	23 (3.8)	3 (4.9)	15 (15.5)^**^
BMI ≥ 25 (kg/m^2^)	329 (54.7)	26 (42.6)	14 (14.4)^**°°^
BMI ≥ 30 (kg/m^2^)	138 (22.9)	6 (9.8)^**^	3 (3.1)^**^
Abdominal Obesity	319 (53.0)	11 (28.2)^**^	19 (26.0)^**^
male AO distribution	196 (32.6)	1 (6.7)^*^	0 (0.0)^**^
female AO distribution	423 (70.4)	10 (41.7)^**^	19 (48.7)^**^

Data are expressed as *n* (and proportion); comparison with GGP (Guadeloupean general population): ^*^p < 0.05; ^**^p < 0.01; comparison with SCC population: ^°°^*p* < 0.01; ^a^GGP data issued from reference (19); AO (abdominal obesity) if waist > 94 cm for males or > 80 cm for females

### Biology

#### Lipid profiles

SCC patients exhibited intermediate mean values for TC, LDL-C, HDL-C and Non HDL-C, higher than those detected in SCA patients (*p* < 0.01 for all, except for Non HDL-C, *p* < 0.05), but lower than those detected in GGP (*p* < 0.01) (Table 1). SCC patients also had higher apoA level (*p* < 0.01) than SCA patients, without any difference observed for apoB level. SCC TG levels were not different from GGP’s, whereas TG levels in SCA were higher than that detected in SCC patients (*p* < 0.05) and GGP (*p* < 0.01). In both sickle genotypes, TG values remained within the normal range (0.97 g/L ± 0.39) (Table 1). Males SCC and SCA patients exhibited lower values than females for TC, LDL-C, Non HDL-C, HDL-C, and apoA levels (p < 0.01, data not shown), and a similar trend was detected for apoB levels (*p* = 0.059). Only TG levels and TG level ≥ 1.50 g/l were found independent from patients’ gender and age in SCC and SCA genotypes (data not shown).

### Focus on TGL, BMI, nutritional status and fasting glycemia

TG level was positively correlated with BMI in SCC (*r* = 0.330, *p* = 0.01) but not in SCA patients, for whom TG level was also independent of the undernutrition and overweight classes. In both sickle genotypes, TG level was independent of waist, WHR and abdominal obesity (data not shown), and fasting glycemia. Moreover, SCA and SCC patients presented both similar and lower fasting glycemia levels than those detected in GGP.

### Anemia, hemolysis, hydroxyurea (HU) and lipids

As expected, both anemia (Hb) and hemolysis (hemolytic component index) were more severe in SCA than in SCC patients (Table 1, *p* < 0.01 for both). Though, neither hemolysis rate in both genotypes, nor HU treatment in SCA patients, impacted the level of any lipid variable (data not shown).

### Associations between lipids, anthropometry and ACS, VOC and OTN

Too few ACS and hospitalized VOC have been reported in SCC patients to allow statistical analysis of these complications in this group (Table 3). Thus, OTN was the only complication analyzed in SCC patients. OTN in SCC and ACS, VOC and OTN in SCA were not affected by gender, BMI, BMI classes, WHR (excepted for OTN in SCC patients), abdominal obesity, TC, LDL-C, HDL-C, apoA or apoB levels or apoB/apoA ratio. SCA patients with TG level higher than 1.50 g/l exhibited more frequently positive histories of ACS than those with lower TG values (*p* < 0.05). In SCA patients with a positive history of either VOC or OTN, we detected as expected higher hematocrit (*p* < 0.05 and *p* < 0.01 respectively), and in case of positive history of OTN, a lower hemolytic component index (*p* < 0.01). However, no association was detected between past history of these complications and lipid levels. In SCC patients, positive history of OTN was also more frequently encountered, when Non HDL-C level > 1.30 g/l (*p* < 0.05) (Table 3).

**Table 3:** Lipid levels of SCA and SCC patients according to ACS, CVO and OTN history

	SCA						SCC	
Characteristics and lipid levels	ACS *n* = 9	NoACS *n* = 88	VOC *n* = 14	NoVOC *n* = 83	OTN *n* = 30	NoOTN *n* = 67	OTN *n* = 15	NoOTN *n* = 47
Female sex	4 (44.4)	50 (56.8)	8 (57.1)	46 (55.4)	19 (63.3)	35 (52.2)	10(66.7)	31 (66.0)
WHR (cm/cm)	0.88 ± 0.02	0.86 ± 0.05	0.87 ± 0.05	0.86 ± 0.05	0.87 ± 0.05	0.86 ± 0.05	0.91 ± 0.04	0.84^*^ ± 0.07
Hematocrit (% ± SD)	23.6 ± 3.2	23.6 ± 3.7	25.7 ± 3.0	23.2^*^ ± 3.7	25.25 ± 3.04	22.85^**^ ± 3.69	30.33 ± 2.65	30.90 ± 2.98
Hemolytic Component	−0.05 ± 1.11	0.01 ± 0.99	− 0.46 ± 0.68	0.07 ± 1.02	− 0.35 ± 0.61	0.16 ^**^ ± 1.10	−0.07 ± 1.09	0.02 ± 0.98
TC (g/l)	1.25 ± 0.30	1.21 ± 0.30	1.23 ± 0.26	1.21 ± 0.30	1.25 ± 0.30	1.20 ± 0.29	1.44 ± 0.32	1.39 ± 0.32
HDL-C (g/l)	0.42 ± 0.13	0.36 ± 0.09	0.38 ± 0.09	0.36 ± 0.10	0.38 ± 0.09	0.36 ± 0.10	0.47 ± 0.14	0.44 ± 0.11
ApoA (g/l)	1.22 ± 0.28	1.09 ± 0.19	1.15 ± 0.16	1.10 ± 0.21	1.15 ± 0.21	1.11 ± 0.20	1.32 ± 0.27	1.22 ± 0.16
Non HDL-C > 1.30 (g/l)	1 (11.1)	5 (5.7)	1 (7.1)	5 (6.0)	3 (10.0)	3 (4.5)	4 (28.6)	3 ^*^(6.5)
LDL-C (g/l)	0.64 ± 0.20	0.66 ± 0.24	0.64 ± 0.21	0.66 ± 0.24	0.68 ± 0.25	0.64 ± 0.23	0.83 ± 0.23	0.78 ± 0.24
ApoB (g/l)	0.58 ± 0.17	0.61 ± 0.19	0.57 ± 0.16	0.61 ± 0.19	0.63 ± 0.19	0.59 ± 0.18	0.69 ± 0.20	0.65 ± 0.19
TG (g/l)	1.06 ± 0.57	0.96 ± 0.37	1.03 ± 0.53	0.96 ± 0.37	0.96 ± 0.37	0.97 ± 0.40	0.80 ± 0.49	0.80 ± 0.42
TG ≥ 1.50 g/l	3 (33.3)	6 ^*^(6.9)	3 (21.4)	6 (7.3)	3 (10.0)	6 (9.1)	1 (6.7)	3 (6.5)

Values represent *n* (and proportion) or mean result ± standard deviation (SD), unless otherwise indicated. Significance: ^*^p < 0.05; ^**^p < 0.01. ACS: acute chest syndrome positive history; NoACS: absence of ACS history; VOC: hospitalized vaso occlusive crisis positive history; NoVOC: absence of VOC history; OTN: Osteonecrosis positive history; NoOTN, absence of OTN history; WHR (waist over hips ratio), TC (total cholesterol), HDL-C (high density lipoprotein-cholesterol), LDL-C (low density lipoprotein- cholesterol), Non HDL-C (Non HDL-cholesterol); ApoA (apolipoprotein A), ApoB (apolipoprotein B), TG (triglycerides)

## Discussion

In contrast to SCA, the plasma lipid profile of SCC patients has been poorly described up to now [7, 10, 16]. This study clearly revealed two very different SCC and SCA lipid profiles, both of them being distinct from their GGP’s. In addition, we described several associations between sickle genotypes and anthropometric phenotypes, as well as between lipid levels and sickle cell complications.

### Distinct lipids profiles in SCA and SCC patients

In agreement with previous studies, our results showed lower lipids values in SCA than in healthy individuals [6], and lower lipids and apoA and apoB levels in males than in females [6, 11]. This present study extends this gender effect to SCC patients. Moreover, if SCC lipids profile presents intermediate values between GGP and SCA, the distinction between SCA and SCC lipid profiles is partially due to higher TG levels in SCA, with unexpected similar values of SCA apoB levels than in SCC. This observation is consistent with the presence of high levels of very low density lipoproteins in SCA [16, 28].

### TG and anthropometric measurements

SCA and SCC patients’ TG levels were both found unexpectedly independent of fasting glycemia. For both genotypes, similar values of fasting glycemia, significantly lower than in GGP, were detected, suggesting undernutrition status [29]. In pathophysiological contexts other than SCD, undernutrition, also explored by BMI class, was reported to modify lipid profiles, with higher TG and lower HDL-C levels in moderately and severely undernourished children, as a mean of adaptation to chronic malnutrition [30]. In agreement with the present study, SCA patients have been reported throughout the world to be more frequently affected by under- or malnutrition [31, 32] compared to SCC patients. Hence, we report for the first time to our knowledge, that TG and TG level ≥ 1.50 g/l remained independent of this so-defined BMI-undernutrition class in SCA patients.

Aside from SCD, adiposity is a significant determinant of both plasma TG and HDL-C levels [31]. TG level is indeed known to increase with both BMI and abdominal obesity [33–36]. In SCD populations, only Zorca et al. concluded that BMI was a “slightly weak but significant predictor of SCD TG level” [7], without reporting any data on abdominal obesity.

In this study, we detected few overweight SCA patients, whereas abdominal obesity was observed both in overweight patients and those with normal BMI. However, no link was detected between TG level and both BMI and abdominal obesity. This normal BMI with abdominal obesity phenotype had been previously reported in a study exclusively dedicated to SCA adult female gender [37]. Our study also explored adult male gender and, for the first time, revealed a striking absence of abdominal obesity in almost all the male patients of both genotypes. This observation may result from the shift of female abdominal obesity to lower levels in SCA and SCC cohorts, compared to the 70.4%+/− 2.5 abdominal obesities in women versus 32.6%+/− 2.5 in males reported in GGP [21]. However, despite having a low or normal BMI, abdominal obesity still remains a source of pro-inflammatory substances, among which are TG-rich lipoproteins lipolysis products [38]. These molecules may increase oxidative stress and endothelial cell inflammation, but this hypothesis remains to be tested.

We also showed for the first time in SCA patients, that TG level was not affected by age, gender, BMI, BMI-nutritional status, waist, WHR or abdominal obesity, and confirmed that TG level was positively correlated with BMI only in SCC patients.

### TG, lipid levels and clinical complications

In contrast to Zorca et al. study [7], TG levels were not associated with hemolysis in our two patients’ cohorts. Otherwise, HU, the only available treatment given to reduce the number and the severity of complications in SCA, did not influence either TG, concordantly with literature [7], or lipoproteins, apoA or apoB levels, as shown by this study.

Apart from TG level ≥ 1.50 g/L, no other lipid association was isolated by this study for ACS history in SCA patients, with too few SCC patients with ACS history to be analyzed.

Higher hematocrit levels were reported in SCA patients with past history of VOC [25], as shown in our study. In addition, both apoA and HDL-C were described to decrease during VOC in SCA [39, 40]. Our analysis, performed only at steady state, did not detect any difference in their levels in relation with clinical history of VOC, advocating for a transient decrease or increase of these lipid levels accordingly to the clinical status of the patients.

Non-traumatic osteonecrosis of the femoral head is a source of major disabling for SCD patients. Various risk factors were previously identified, among which, as confirmed in our study, higher hematocrit in SCA for OTN, and lower hemolysis in SCC patients, respectively [23, 41]. Several lipid risk factors of osteonecrosis, so far unexplored in SCD, were also highlighted in the literature. Among them, high levels of TC [42, 43], apoB/apoA ratio [44], TG [43, 45], and Non HDL-C have been described [43]. We did not detect any association between SCC or SCA OTN history and TC, LDL-C, TG, apoA or apoB levels, or apoB/apoA ratio. However, our study showed that SCC patients with OTN history presented both higher WHR and Non HDL-C levels than patients without, suggesting a more atherogenous profile in the former patients [46]. Our data suggest that Non HDL-C level ≥ 1.30 g/l could be a biomarker associated with SCC OTN history.

Limits of our study are those of a cross-sectional one that could not represent individual daily lipid profiles [47], with known normal variation of fasting TG level, and include the size of the two patient cohorts. These primary statements have to be confirmed by larger prospective studies, which should include evaluation of diet uptake, a known major modulator of plasma lipid profile.

## Conclusions

Our retrospective study revealed two different lipid profiles in SCC and SCA Guadeloupian cohorts, also different from their GGP. Our sickle genotype-based analysis revealed that SCA mean TG level was higher compared to both SCC and GGP, and furthermore was independent of HU treatment, hemolysis, age, gender, BMI, abdominal obesity and clinical nutritional status. Unless for TG level, the study confirmed a female gender effect on lipids, apoA and apoB levels in both sickle cell genotypes, where a similar abdominal obesity was observed, almost exclusively encountered in females. The search for lipid biomarkers of SCD complications revealed that TG level ≥ 1.50 g/l and Non HDL-C level ≥ 1.30 g/l were linked with more frequent positive history of ACS in SCA patients and more frequent positive history of OTN in SCC patients, respectively.

## Abbreviations

ACS: Acute chest syndrome
AO: Abdominal obesity following International Diabetes Federation definition
Apo: Apolipoprotein
AST: Aspartate amino-transferase
BIL: Total bilirubin
BMI: Body mass index
ESC/EAS: European Society of Cardiology/European Atherosclerosis Society
FG: Fasting glycemia
GGP: Guadeloupian general population
Hb: Hemoglobin
HbS: hemoglobin S
HDL-C: HDL-Cholesterol
HU: Hydroxyurea
LDH: Lactate dehydrogenase
LDL-C: LDL-Cholesterol
NHANES: National Health and Nutrition Examination Surveys
NoACS: Absence of acute chest syndrome history
Non HDL-C: Non HDL-Cholesterol
NoOTN: Absence of osteonecrosis history
NoVOC: Absence of hospitalized vaso-occlusive crisis history
OTN: Osteonecrosis
PLT: Platelet
RBCs: Red blood cells count
RET: Reticulocytes
SCA: Sickle cell anemia
SCC: Sickle cell SC disease
SCD: Sickle cell disease
SD: Standard deviation
TC: Total cholesterol
TG: Triglycerides
VOC: Vaso-occlusive crisis
WBC: White blood cell counts
WHR: Waist-to-hip ratio.

## References

[1] Rees DC, Williams TN, Gladwin MT (2010). Sickle-cell disease. Lancet.

[2] Schnog JB, Duits AJ, Muskiet FAJ, ten Cate H, Rojer RA, Brandjes DPM (2004). Sickle cell disease; a general overview. Neth J Med.

[3] Lionnet F, Hammoudi N, Stojanovic KS, Avellino V, Grateau G, Girot R, Haymann JP (2012). Hemoglobin sickle cell disease complications: a clinical study of 179 cases. Haematologica.

[4] Lemonne N, Lamarre Y, Romana M, Hardy-Dessources MD, Lionnet F, Waltz X, Tarer V, Mougenel D, Tressières B, Lalanne-Mistrih M-L, Etienne-Julan M, Connes P (2014). Impaired blood rheology plays a role in the chronic disorders associated with sickle cell-hemoglobin C disease. Haematologica.

[5] Sasaki J, Waterman MR, Cottam GL (1986). Decreased apolipoprotein A-I and B content in plasma of individuals with sickle cell anemia. Clin Chem.

[6] Rahimi Z, Merat A, Haghshenass M, Madani H, Rezaei M, Nagel RL (2006). Plasma lipids in Iranians with sickle cell disease: hypocholesterolemia in sickle cell anemia and increase of HDL-cholesterol in sickle cell trait. Clin Chim Acta.

[7] Zorca S, Freeman L, Hildesheim M, Allen D, Remaley AT, Taylor JG, Kato GJ (2010). Lipid levels in sickle-cell disease associated with haemolytic severity, vascular dysfunction and pulmonary hypertension. Br J Haematol.

[8] Seixas MO, Rocha LC, Carvalho MB, Menezes JF, Lyra IM, Nascimento VML, Couto RD, Atta AM, Reis MG, Goncalves MS (2010). Levels of high-density lipoprotein cholesterol (HDL-C) among children with steady-state sickle cell disease. Lipids Health Dis.

[9] Shores J, Peterson J, VanderJagt D, Glew RH (2003). Reduced cholesterol levels in African-American adults with sickle cell disease. J Natl Med Assoc.

[10] Buchowski MS, Swift LL, Akohoue SA, Shankar SM, Flakoll PJ, Abumrad N (2007). Defects in postabsorptive plasma homeostasis of fatty acids in sickle cell disease. J Parenter Enter Nutr.

[11] Yuditskaya S, Tumblin A, Hoehn GT, Wang G, Drake SK, Xu X, Ying S, Chi AH, Remaley AT, Shen RF, Munson PJ, Suffredini AF, Kato GJ (2009). Proteomic identification of altered apolipoprotein patterns in pulmonary hypertension and vasculopathy of sickle cell disease. Blood.

[12] Aleluia MM, da Guarda CC, Santiago RP, Fonseca TC, Neves FI, de Souza RQ, Farias LA, Pimenta FA, Fiuza LM, Pitanga TN, Ferreira JR, Adorno EV, Cerqueira BA, Gonçalves MS (2017). Association of classical markers and establishment of the dyslipidemic sub-phenotype of sickle cell anemia. Lipids Health Dis.

[13] Lamarre Y, Lalanne-Mistrih M-L, Romana M, Lemonne N, Mougenel D, Waltz X, Tressières B, Etienne-Julan M, Tarer V, Hardy-Dessources MD, Connes P (2013). Male gender, increased blood viscosity, body mass index and triglyceride levels are independently associated with systemic relative hypertension in sickle cell anemia. PLoS One.

[14] Ataga KI, Hinderliter A, Brittain JE, Jones S, Xu H, Cai J, Kim S, Pritchard KA, Hillery CA (2015). Association of pro-inflammatory high-density lipoprotein cholesterol with clinical and laboratory variables in sickle cell disease. Hematology.

[15] Ji X, Feng Y, Tian H, Meng W, Wang W, Liu N, Zhang J, Wang L, Wang J, Gao H (2016). The mechanism of Proinflammatory HDL generation in sickle cell disease is linked to cell-free hemoglobin via Haptoglobin. PLoS One.

[16] Ephraim RK, Adu P, Ake E, Agbodzakey H, Adoba P, Cudjoe O, Agoni C (2016). Normal non-HDL cholesterol, low Total cholesterol, and HDL cholesterol levels in sickle cell disease patients in the steady state: a case-control study of Tema Metropolis. J Lipids.

[17] Pi-Sunyer FX, Becker DM, Bouchard C, Carleton RA, Colditz GA, Dietz WH, Foreyt JP, Garrison RJ, Grundy SC, Hansen BC, Higgins M, Hill JO, Howard BV, Klesges RC, Kuczmarski RJ, Kumanyika S, Legako RD, T. Prewitt E, Rocchini AP, Smith PL, Snetselaar LG, Sowers JR, Weintraub M, Williamson DF, G. WilsonT. Executive summary: clinical guidelines on the identification, evaluation, and treatment of overweight and obesity in adults --the evidence report. National Institutes of Health. Obes Res. 1998;6(Suppl 2):51S–209S. 9813653

[18] Amato MC, Guarnotta V, Giordano C (2013). Body composition assessment for the definition of cardiometabolic risk. J Endocrinol Investig.

[19] Alberti KG, Zimmet P, Shaw J (2005). IDF epidemiology task force consensus group. The metabolic syndrome--a new worldwide definition. Lancet.

[20] Foucan L, Kangambega P, Ekouévi DK, Rozet J, Bangou-Brédent J (2000). Lipid profile in an adult population in Guadeloupe. Diabetes Metab..

[21] Daigre JL, Atallah A, Boissin JL, Jean-Baptiste G, Kangambega P, Chevalier H, Balkau B, Smadja D (2012). The prevalence of overweight and obesity, and distribution of waist circumference, in adults and children in the French overseas territories: the PODIUM survey. Diabetes Metab.

[22] Mukisi-Mukaza M, Elbaz A, Samuel-Leborgne Y, Kéclard L, Le Turdu-Chicot C, Christophe-Duchange E, Mérault G (2000). Prevalence, clinical features, and risk factors of osteonecrosis of the femoral head among adults with sickle cell disease. Orthopedics.

[23] Mukisi-Mukaza M, Saint Martin C, Etienne-Julan M, Donkerwolcke M, Burny ME, Burny F (2011). Risk factors and impact of orthopaedic monitoring on the outcome of avascular necrosis of the femoral head in adults with sickle cell disease: 215 patients case study with control group. Orthop Traumatol Surg Res.

[24] Lamarre Y, Romana M, Waltz X, Lalanne-Mistrih M-L, Tressières B, Divialle-Doumdo L, Hardy-Dessources MD, Vent-Schmidt J, Petras M, Broquere C, Maillard F, Tarer V, Etienne-Julan M, Connes P (2012). Hemorheological risk factors of acute chest syndrome and painful vaso-occlusive crisis in children with sickle cell disease. Haematologica.

[25] Ballas SK, Larner J, Smith ED, Surrey S, Schwartz E, Rappaport EF (1988). Rheologic predictors of the severity of the painful sickle cell crisis. Blood.

[26] Nouraie M, Lee JS, Zhang Y, Kanias T, Zhao X, Xiong Z, Oriss TB, Zeng Q, Kato GJ, Gibbs JS, Hildesheim ME, Sachdev V, Barst RJ, Machado RF, Hassell KL, Little JA, Schraufnagel DE, Krishnamurti L, Novelli E, Girgis RE, Morris CR, Rosenzweig EB, Badesch DB, Lanzkron S, Castro OL, Goldsmith JC, Gordeuk VR, Gladwin MT, Walk-PHASST Investigators and Patients (2013). The relationship between the severity of hemolysis, clinical manifestations and risk of death in 415 patients with sickle cell anemia in the US and Europe. Haematologica.

[27] Catapano AL, Reiner Z, De Backer G, Graham I, Taskinen MR, Wiklund O, Agewall S, Alegria E, Chapman MJ, Durrington P, Erdine S, Halcox J, Hobbs R, Kjekshus J, Filardi PP, Riccardi G, Storey RF, Wood D (2011). ESC/EAS guidelines for the management of dyslipidaemias: the task force for the management of dyslipidaemias of the European Society of Cardiology (ESC) and the European atherosclerosis society (EAS). Eur Heart J.

[28] Ozturk OH, Can Y, Yonden Z, Motor S, Oktay G, Kaya H, Aslan M (2013). Lipoprotein subfraction profile and HDL-associated enzymes in sickle cell disease patients. Lipids.

[29] Karl JP, Smith TJ, Wilson MA, Bukhari AS, Pasiakos SM, McClung HL, Liberman HR (2016). Altered metabolic homeostasis is associated with appetite regulation during and following 48-h of severe energy deprivation in adults. Metabolism.

[30] Veiga GR, Ferreira HS, Sawaya AL, Calado J, Florêncio TM (2010). Dyslipidaemia and undernutrition in children from impoverished areas of Maceió, state of Alagoas, Brazil. Int J Environ Res Public Health.

[31] Singhal A, Davies P, Sahota A, Thomas PW, Serjeant GR (1993). Resting metabolic rate in homozygous sickle cell disease. Am J Clin Nutr.

[32] Prasad AS (1997). Malnutrition in sickle cell disease patients. Am J Clin Nutr.

[33] Cole CB, Nikpay M, Lau P, Stewart AFR, Davies RW, Wells GA, Dent R, McPherson R (2014). Adiposity significantly modifies genetic risk for dyslipidemia. J Lipid Res.

[34] Oka R, Kobayashi J, Miura K, Nagasawa S, Moriuchi T, Hifumi S, Miyamoto S, Kawashiri MA, Nohara A, Inazu A, Takeda Y, Mabuchi H, Yagi K, Yamagishi M (2009). Difference between fasting and nonfasting triglyceridemia; the influence of waist circumference. J Atheroscler Thromb.

[35] Sahade V, França S, Adan LF (2013). The influence of weight excess on the postprandial lipemia in adolescents. Lipids Health Dis.

[36] Fan H, Li X, Zheng L, Chen X, Lan Q, Wu H, Ding X, Qian D, Shen Y, Yu Z, Fan L, Chen M, Tomlinson B, Chan P, Zhang Y, Liu Z (2016). Abdominal obesity is strongly associated with cardiovascular disease and its risk factors in elderly and very elderly community-dwelling Chinese. Sci Rep.

[37] Woods KF, Ramsey LT, Callahan LA, Mensah GA, Litaker MS, Kutlar A, Barbeau P, Gutin B (2001). Body composition in women with sickle cell disease. Ethn Dis.

[38] Wang L, Gill R, Pedersen TL, Higgins LJ, Newman JW, Rutledge JC (2009). Triglyceride-rich lipoprotein lipolysis releases neutral and oxidized FFAs that induce endothelial cell inflammation. J Lipid Res.

[39] Monnet PD, Kane F, Konan-Waidhet D, Akpona S, Kora J, Diafouka F, Sess D, Sangare A, Yapo AE (1996). Evaluation of atherogenic risk in homozygous sickle cell disease: study of lipid and apolipoprotein AI and B plasma levels. Bull Soc Pathol Exot.

[40] Monnet D, Edjeme NE, Ndri K, Hauhouot-Attoungbre ML, Ahibo H, Sangare A, Yapo AE (2002). Lipoprotein (a) and acute phase inflammation proteins in homozygous sickle cell disease. Ann Biol Clin (Paris).

[41] Milner PF, Kraus AP, Sebes JI, Sleeper LA, Dukes KA, Embury SH, Bellevue R, Koshy M, Moohr JW, Smith J (1991). Sickle cell disease as a cause of osteonecrosis of the femoral head. N Engl J Med.

[42] Moskal JT, Topping RE, Franklin LL (1997). Hypercholesterolemia: an association with osteonecrosis of the femoral head. Am J Orthop (Belle Mead NJ).

[43] Zhao DW, Yu M, Hu K, Wang W, Yang L, Wang BJ, Gao XH, Guo YM, Xu YQ, Wei YS, Tian SM, Yang F, Wang N, Huang SB, Xie H, Wei XW, Jiang HS, Zang YK, Ai J, Chen YL, Lei GH, Li YJ, Tian G, Li ZS, Cao Y, Ma L (2015). Prevalence of nontraumatic osteonecrosis of the femoral head and its associated risk factors in the Chinese population: results from a nationally representative survey. Chin Med J.

[44] Miyanishi K, Yamamoto T, Irisa T, Noguchi Y, Sugioka Y, Iwamoto Y (1999). Increased level of apolipoprotein B/apolipoprotein A1 ratio as a potential risk for osteonecrosis. Ann Rheum Dis.

[45] Kuroda T, Tanabe N, Wakamatsu A, Takai C, Sato H, Nakatsue T, Wada Y, Nakano M, Narita I (2015). High triglyceride is a risk factor for silent osteonecrosis of the femoral head in systemic lupus erythematosus. Clin Rheumatol.

[46] Bergmann K (2010). Non-HDL cholesterol and evaluation of cardiovascular disease risk. E J I F C C.

[47] Jaskolowski J, Ritz C, Sjödin A, Astrup A, Szecsi PB, Stender S, Hjorth MF (2017). Weekday variation in triglyceride concentrations in 1.8 million blood samples. J Lipid Res.

